# Electrochemical Corrosion and Catalysis Dynamics of Tin Oxide during Water Oxidation

**DOI:** 10.1021/acscatal.5c04461

**Published:** 2025-10-28

**Authors:** Rayan Alaufey, Lingyan Zhao, Christina Lents, Brianna Markunas, Adam D. Walter, Qin Wu, John A. Keith, Maureen Tang

**Affiliations:** † Department of Chemical and Biological Engineering, 6527 Drexel University, Philadelphia 19104, Pennsylvania, United States; ‡ Department of Chemical and Petroleum Engineering, 6614 University of Pittsburgh, 3700 O’Hara Street, Pittsburgh, Pennsylvania 15261, United States; § Department of Material Science and Engineering, Drexel University, Philadelphia 19104, Pennsylvania, United States; ∥ Center for Functional Nanomaterials, 8099 Brookhaven National Laboratory, Upton, New York 11973, United States

**Keywords:** tin oxide, corrosion, catalysis, interfacial networks, defects

## Abstract

Metal oxide corrosion severely limits anodic electrocatalysis, particularly at high potentials in acidic environments, where degradation pathways remain poorly defined. This study establishes explicit connections between corrosion and electrocatalysis on tin oxide during water oxidation by examining the roles of lattice defects, reactive oxygen species, interfacial pH variations, and speciation of corroded tin in acid. We first demonstrate the presence of structural defects such as oxygen vacancies and substoichiometric Sn­(II) species by integrating electron paramagnetic resonance spectroscopy, ultraviolet photoelectron spectroscopy, and Mott–Schottky analysis. Kohn–Sham density functional theory calculations reveal that explicit water structures thermodynamically stabilize reaction intermediates and lower reaction overpotentials. Moreover, we propose that water dissociation leads to hydrogen-bonding networks formed by H* and OH* intermediates, which may span the entire catalyst surface and decrease the interfacial pH to drive corrosion. In contrast, the electrochemical generation of reactive oxygen species is shown to play a minor role in catalyst corrosion during water oxidation using inductively coupled plasma mass spectrometry coupled with selective chemical scavengers. Square-wave voltammetry combined with rotating ring-disk electrodes is used to reveal that under open-circuit conditions, only Sn­(IV) cations chemically dissolve from tin oxide, while both Sn­(IV) and Sn­(II) species electrochemically corrode during water oxidation. Our results unveil a dynamic and complicated interplay between corrosive and catalytic pathways on metal oxide electrocatalysts: a decrease in interfacial pH due to water oxidation exacerbates Sn­(II)/Sn­(IV) corrosion. Subsequently, the electrochemical corrosion of Sn­(II)/Sn­(IV) facilitates product formation from lattice oxygen, while the redeposition of corroded Sn­(II) as Sn­(IV) can enable oxygen exchange with water. By elucidating the roles of defects and interfacial chemistry, this work provides a roadmap for engineering improved electrocatalysts that balance activity and stability, a critical step toward scalable and durable energy technologies.

## Graphical abstract

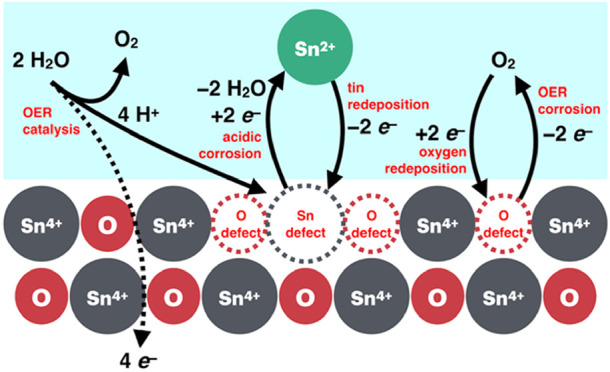

## 1 Introduction

Catalysts are defined as substances that accelerate chemical reactions without undergoing a permanent chemical change,^1^ but this definition falls short when applied to metal oxides employed for electrochemical oxidation reactions. Many oxides undergo irreversible alterations, including chemical dissolution, galvanic corrosion, and other oxidative pathways, that degrade their structure and performance.^2–7^ In this work, we distinguish these degradation modes. Galvanic corrosion of metal oxides is defined as an electrochemical degradation process driven by oxidative charge transfer within the oxide framework, which may involve oxidation of lattice oxygen anions (2O^2–^ → O_2_ + 4 e^–^) from fully occupied 2p orbitals, driving metal cation release, and/or direct oxidation of metal cations to higher valence states (M^n+^ → M^(n+m)+^ + m e^–^) that favor dissolution through contraction of the ionic radius. In both cases, the oxidative step is directly coupled to product liberation into the electrolyte and is accompanied by Faradaic electron transfer.^8,9^ Chemical dissolution, in contrast, is defined as an acid–base process involving direct proton exchange between the catalyst surface and the electrolyte, operating independently of external circuitry. A thorough understanding of all of these pathways is essential because they collectively contribute to catalyst instability. This instability, particularly under the demanding conditions of electrochemical oxidation reactions, represents a major bottleneck for the efficiency and durability of energy conversion technologies.^10,11^

Past research has focused on the degradation of highly active oxides, such as iridium oxide and ruthenium oxide, which have exceptional oxygen evolution reaction (OER) activity.^12,13^ These materials are pivotal for sustainable, green hydrogen production via water electrolysis. However, OER itself has been suggested to induce galvanic corrosion,^9,14–16^ leading to the irreversible destruction of these catalysts. Intriguingly, corrosion limits catalyst lifetimes and complicates mechanistic studies, as structural and compositional evolution obscures the relationship between intrinsic material properties and performance. Dissolved metal cations have been proposed to act as homogeneous intermediates in the OER mechanism, creating a complex interplay between electrocatalysis and corrosion.^17^ Unlike conventional active OER catalysts, materials such as titanium oxide, lead oxide, and tin oxide, which are often considered catalytically “inert” for OER, have been studied less extensively.^18,19^ These materials can oxidize water, albeit at substantially greater overpotentials (i.e., much higher applied potentials) compared to active OER catalysts. This property makes them suitable for specialized applications such as water oxidation to reactive oxygen species (ROS), including ozone, hydrogen peroxide, and hydroxyl radicals, as well as for wastewater treatment, and electrosynthesis.^20,21^ Their intrinsic properties and degradation mechanisms, however, have been less investigated. Tin oxide has several convenient properties (e.g., well-defined crystallinity, predominantly existing in the tetragonal phase, alongside its low toxicity, affordability, tunable electronic properties, and relevance in electrocatalysis)^5,22^ that make it a suitable model system for other “OER-inert” metal oxides. Therefore, in this study, we use tin oxide to elucidate pathways that lead to inert metal oxide corrosion and examine how nonidealities, such as nonstoichiometry and interfacial water, influence the dynamic interplay between water oxidation and catalyst degradation.

Binninger et al.^9^ originally proposed that chemical dissolution of sparingly soluble oxides inherently enables oxygen evolution via galvanic corrosion, and we further interrogate this idea. Their model posits that O_2_ can be generated directly from lattice O, directly coupling the OER to galvanic corrosion. This analysis suggests that achieving OER conditions in acid, where nearly all metal oxides dissolve, inevitably induces galvanic corrosion, establishing a thermodynamic stability limit for metal oxide electrocatalysts. Our prior work attempted to extend this analysis to study hydrogen peroxide and ozone formation on tin oxide, utilizing mass spectrometry and ^18^O isotopic labeling. Our results demonstrated the direct generation of products from the tin oxide lattice, leading to the irreversible depletion of both oxygen anions and tin cations.^2^ Similar results were obtained by Geiger and colleagues, who demonstrated that oxygen generation using doped tin oxide is coupled to catalyst degradation.^6^ Their work identified galvanic corrosion as a primary mechanism responsible for tin oxide degradation by using online inductively coupled plasma mass spectrometry (ICP-MS) to monitor the onset of catalyst corrosion as a function of the applied potential. However, our quantum chemistry calculations did not corroborate these experimental findings, suggesting that galvanic corrosion of tin oxide should only become feasible at potentials significantly higher than those employed experimentally.^2^ We note that these previous studies all employed doped tin oxide, which exhibits higher electrical conductivity, while our present work employs undoped SnO_2_. This distinction is particularly important because prior studies have shown that conductivity in tin oxide is directly correlated with electrochemical activity, a relationship that often comes at the expense of stability.^3,23^ Our work, therefore, seeks to isolate the intrinsic reactivity and degradation behavior of the undoped catalyst.

Furthermore, a critical gap in previous studies is the implicit assumption that the metal oxide catalyst is an ideal material that can be perceived as more or less perfectly stoichiometric, free from defects, and with no adsorbed water on its surface.^24–26^ However, this idealization does not reflect the complexities of real-world electrocatalysts.^3,27,28^ For example, substoichiometric Sn­(II) cations and oxygen vacancies (O_vac_) in tin oxide are known to exist and govern electronic behavior.^18,19^ Compounding this complexity, adsorbed water layers under operational conditions may actively mediate both catalytic and corrosive processes, creating intertwined degradation pathways. By moving beyond idealized models, we aim to resolve discrepancies between prior experimental and computational findings and provide insights into the degradation mechanisms of real-world electrocatalysts, guiding the design of more stable and efficient systems for energy applications.

## 2 Methods

### 2.1 Synthesis of Tin Oxide Powder

Anhydrous tin­(II) chloride (SnCl_2_, 99.5%, Sigma-Aldrich) and ethanol (C_2_H_5_OH, 200 proof, Decon Laboratories) were used as received without any additional purification steps. To prepare the precursor solution, 190 mg of SnCl_2_ was weighed and dissolved in 50 mL of ethanol with constant magnetic stirring. The mixture was stirred for 5 min, ensuring complete dissolution of the salt and the formation of a homogeneous, colorless solution. Following dissolution, 250 μL of ammonium hydroxide (NH_4_OH, 30% v/v, Sigma-Aldrich) was added dropwise to the precursor solution under continuous stirring. The gradual addition of the base resulted in an immediate turbidity change, forming a cloudy suspension indicative of hydrolysis and the initial precipitation of tin hydroxide species. The suspension was maintained under stirring for a total of 3 h. Notably, after approximately 1 h of stirring, the initially cloudy mixture gradually redissolved, yielding a clear solution, suggesting the transformation of the intermediate hydroxide precipitate into a soluble complex.

The resulting solution was carefully transferred to an alumina boat and subjected to drying at 65 °C until complete solvent evaporation was achieved, leaving behind a solid precursor residue. The dried material was subsequently calcined in a muffle furnace at 250 °C for 3 h under ambient atmosphere. During calcination, the precursor underwent thermal decomposition and oxidation, yielding a yellow tin oxide powder. To ensure the removal of unreacted precursors, residual chlorides, and other impurities, the obtained powder was washed several times with deionized water and ethanol. The washed powder was then dried at room temperature under ambient conditions for 24 h, resulting in the final tin oxide material used for subsequent characterization and electrochemical studies.

### 2.2 Catalyst Ink Preparation

For electrode fabrication, 5 mg of the synthesized tin oxide powder was weighed and dispersed in a solvent/binder mixture consisting of 2.4 mL of isopropyl alcohol (C_3_H_8_O, 99.9%, Sigma-Aldrich), 100 μL of 0.001 M sodium hydroxide (NaOH, Sigma-Aldrich), and 10 μL of Nafion solution (10% v/v, Sigma-Aldrich). The inclusion of sodium hydroxide served to assist in dispersing the oxide particles, while Nafion acted as a polymeric binder to promote adhesion of the catalyst film to the electrode substrate. The resulting suspension was sonicated in a bath sonicator for 30 min to achieve a homogeneous and stable ink, ensuring uniform dispersion of the tin oxide nanoparticles and preventing aggregation. A fresh batch of catalyst ink was prepared immediately prior to the fabrication of each electrode to minimize the variability associated with particle settling or solvent evaporation and to ensure consistent electrode performance across measurements.

### 2.3 Partial Reduction of Tin oxide

For the partial reduction experiments, an aliquot of the sol–gel precursor solution was taken directly and drop-cast onto the surface of a pretreated titanium foil (0.25 cm^2^), eliminating the need for separate powder synthesis and ink preparation steps. The deposited liquid was allowed to evaporate by placing the foil on a silicon wafer maintained at 85 °C for 5 min, promoting controlled solvent removal and uniform film formation. The foil was subsequently transferred to a muffle furnace and calcined for 3 h at either 250 °C, yielding yellow tin oxide, or 1000 °C, yielding white tin oxide. To induce partial reduction, electrodes initially calcined at 250 °C were further annealed in a tube furnace at 250 °C under a forming gas atmosphere (5% H_2_ in Ar) for 1 h, producing dark gray tin oxide. This post-treatment facilitated controlled reduction of Sn^4+^ species, enabling systematic investigation of the effects of the oxidation state on the electrocatalytic behavior of the resulting films.

### 2.4 Material Characterization

X-ray photoelectron spectroscopy (XPS) and ultraviolet photoelectron spectroscopy (UPS) were performed using a Versa Probe 5000 spectrometer (Physical Electronics Inc.) equipped with a monochromatic Al Kα radiation source (200 μm spot size, 25 W power, and 15 kV accelerating voltage). A freshly polished silver foil with a measured, known work function of 4.3 eV was used as the UPS reference. XPS peak fitting and analysis were conducted using CasaXPS software, with the adventitious carbon C 1s peak at 284.8 eV employed as the charge correction reference.

X-ray diffraction (XRD) patterns were collected using a Rigaku Miniflex diffractometer in Bragg–Brentano geometry with a Cu Kα radiation source (λ = 1.54056 Å) to determine the crystalline structure and phase composition of the tin oxide samples. The electrical conductivity of the powders was measured by using the standard four-probe method on an Ossila T2001A3 Hall effect measurement system at a current of 1 mA and room temperature.

Electrochemical impedance spectroscopy (EIS) was carried out in potentiostatic mode using a 10 mV AC voltage perturbation over a frequency range of 10 kHz to 0.1 Hz, with ten points per decade, to probe the interfacial charge-transfer resistance and capacitance of the electrodes. The optical bandgap of the tin oxide samples was determined from ultraviolet–visible (UV–vis) absorption spectra acquired by using a PerkinElmer Lambda 35 spectrophotometer. Finally, electron paramagnetic resonance (EPR) spectra were recorded at room temperature on a Bruker ELEXSYS E500 spectrometer to identify the paramagnetic species and defect states within the oxide lattice.

### 2.5 Electrochemical Measurement and Sn Detection

Electrochemical measurements were carried out using a BioLogic potentiostat in either a standard three-electrode configuration or a four-electrode setup for rotating ring-disk electrode (RRDE) experiments. The working electrode was a glassy carbon disk (*A* = 0.196 cm^2^), which was carefully polished with alumina prior to drop-casting the tin oxide catalyst ink. For RRDE measurements, the ring electrode was composed of gold, platinum, or glassy carbon with no catalyst deposited. A BASi Ag/AgCl reference electrode (3.0 M KCl) and a graphite rod counter electrode were employed in all experiments. The electrolyte consisted of 1 M H_2_SO_4_ (pH = 0), which was continuously purged with argon to maintain an inert environment during pulse voltammetry measurements. During RRDE experiments, the working electrode was rotated at 1600 rpm to ensure a steady flux of reactants to the disk and ring surfaces, while the rotation was halted for square-wave voltammetry (SWV) experiments. All measurements were conducted at room temperature in a four-necked glass cell equipped with dedicated inlets for the working, counter, and reference electrodes as well as argon purging. SWV measurements were performed with a potential step of 5 mV, an amplitude of 10 mV, and a frequency of 10 Hz, conditions initially optimized to maximize amperometric signals from SnCl_4_ and SnCl_2_ in 1 M HCl and then maintained for all other compositions.

To enhance the detection of the small, dilute amounts of tin released from the corroding tin oxide disk, our approach employs a two-stage electrochemical process on the collector ring. An initial 10 min electrodeposition step accumulates tin species from the electrolyte, increasing the analytical signal to detectable levels. The applied potential during this accumulation phase is carefully controlled to differentiate the tin oxidation states. Sn­(IV) cations generated at the disk are collected on the ring via a cathodic potential, causing their reduction to metallic tin. Dissolved Sn­(II) cations can follow two pathways: cathodic electroplating as metallic tin, or anodic collection, where Sn­(II) is oxidized to Sn­(IV) and subsequently hydrolyzes to form a precipitate of tin­(IV) oxide (SnO_2_) on the ring.

Following selective electrodeposition, SWV is applied to the ring to confirm the presence of the deposited Sn and quantify its amount. The SWV sweep is conducted in the potential direction opposite to the initial deposition, enabling oxidation of cathodically plated Sn to Sn­(IV) or reduction of anodically precipitated SnO_2_, thereby generating a clear current signal. This combination of controlled, selective electrodeposition for accumulation and SWV for sensitive detection provides a robust method for identifying and quantifying the specific oxidation states of tin species released during the corrosion of tin oxide on the disk.

For ICP-MS measurements, aqueous samples were diluted to 50 mL with ultrapure water (10× dilution). The samples were introduced into an ICP-QQQ (8900 with SPS 4 autosampler, Agilent, Santa Clara, CA) and analyzed in helium mode. Ultrapure water was used as the blank, and an Sn ICP-MS calibration standard (5190–8544, Agilent Technologies, Santa Clara, USA) was employed to determine the tin concentration.

## 3 Results and Discussion

Accurately modeling the activity of oxide catalysts during water oxidation requires assumptions about their structure and composition. In the case of tin oxide, many treatments have relied on idealized models, assuming perfect stoichiometry, defect-free surfaces, and the absence of interfacial water. These simplifications, while useful, can lead to inconsistencies when compared with experimental observations. To bridge this gap, we first examine the tin oxide catalyst used in this study and demonstrate that it exhibits defects and other nonideal features. Defects in tin oxide are known to be present and are inherently responsible for its electronic properties.^3,29,30^ As a post-transition metal oxide, tin oxide is characterized by a wide bandgap, which limits its electrical conductivity.^31^

To estimate the optical bandgap of the catalyst, we employed a Tauc plot analysis. This approach involves plotting the photon energy dependence of the absorption coefficient and extrapolating the linear portion of the curve to determine the bandgap. As shown in Figure 1A, the Tauc plot reveals an indirect bandgap of 3.90 eV for the tin oxide particles synthesized via sol–gel at 250 °C used in this study. To probe the electronic structure further, we performed UPS. This technique measures the kinetic energy of electrons ejected from a material when irradiated with ultraviolet photons, allowing the direct determination of the valence band maximum relative to the vacuum level. The UPS spectrum in Figure 1B shows that the valence band maximum is located 9.1 eV below the vacuum level, consistent with values reported in the literature.^32,33^ In an ideal, perfectly stoichiometric tin oxide lacking defects, the valence band primarily consists of O2p orbitals, while the conduction band is composed of unoccupied Sn5s orbitals.^34^ The large bandgap suggests insulating behavior at room temperature, as ambient thermal energy is insufficient to excite electrons to the conduction band.^27,28^ However, four-point probe measurements showed a moderate electrical resistivity for tin oxide at 1.69 ± 0.02 Ω·cm, as shown in Table 1, which is in the range of a semiconductor rather than that of a true insulator. This discrepancy underscores the role of structural defects and substoichiometry in enhancing electrical conductivity, which allows tin oxide to be used as an electrocatalyst.

**Figure 1:**
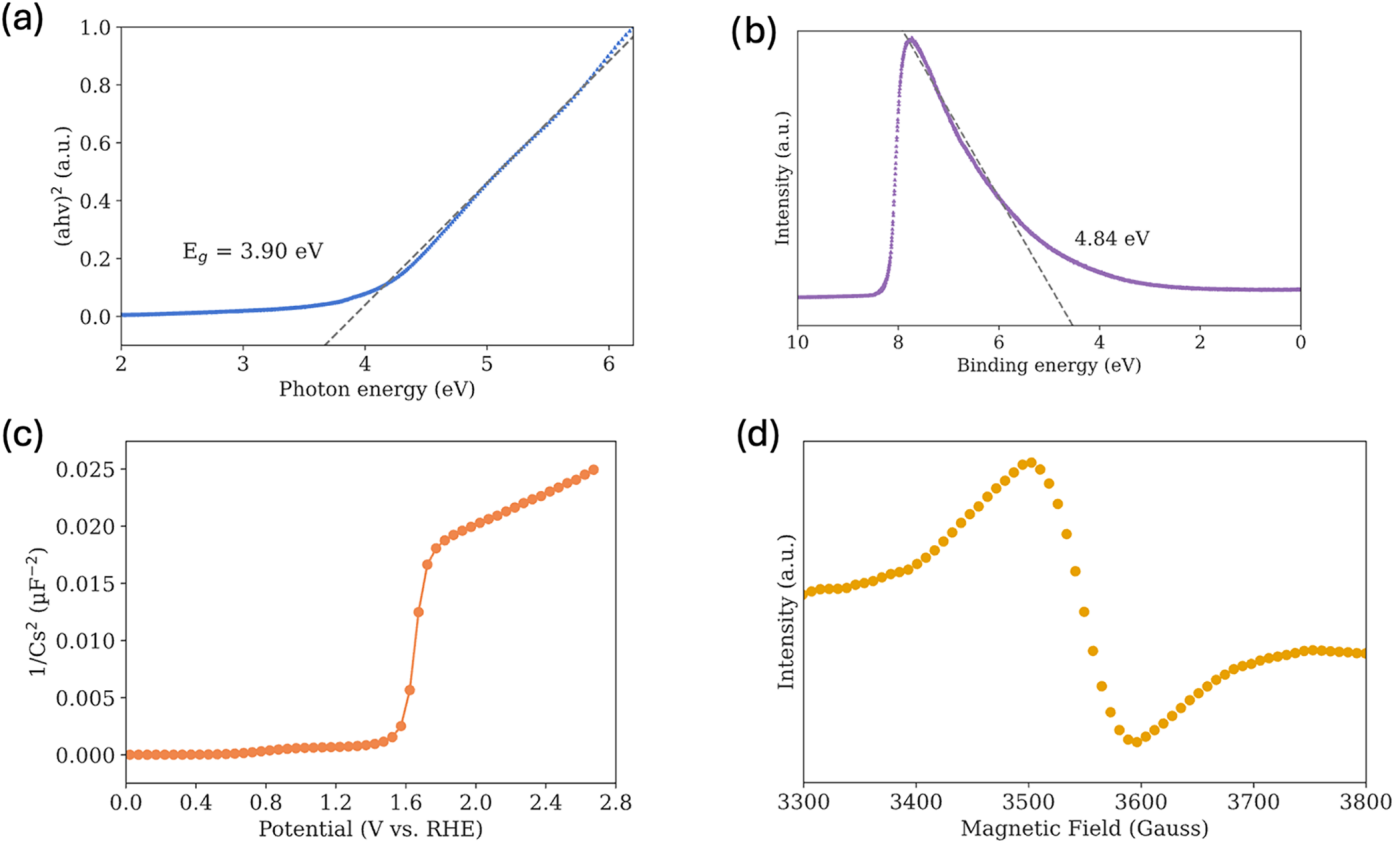
(a) Tauc plot of tin oxide, showing the optical absorption edge and used to determine an indirect bandgap of 3.90 eV. (b) UPS spectrum indicating the position of the valence band maximum relative to the vacuum level, consistent with literature values. (c) Mott–Schottky plot exhibiting a positive slope across the measured potential window, confirming n-type conductivity and the presence of negatively charged electrons as majority carriers. (d) EPR spectrum of tin oxide, revealing the characteristic signal of singly ionized oxygen vacancies, which serve as paramagnetic defect sites influencing electronic properties.

**Table 1:** Electrical Resistivity Data

catalyst	electrical resistivity (Ω·cm)
partially reduced	0.014 ± 0.0025
250 °C	1.69 ± 0.02
1000 °C	182 ± 4.3

To support this claim, we employed Mott–Schottky analysis. This method relates the capacitance of the space-charge region to the applied potential, and the slope of the resulting plot indicates the type of charge carriers present. The Mott–Schottky plot in Figure 1C exhibits a positive slope across the entire studied potential window (0–2.7 V vs RHE), confirming n-type conductivity due to negatively charged electrons.^35,36^ This observation indicates that defects act as localized electron donors within the tin oxide bandgap, consistent with literature.^37,38^ The change in slope of the Mott–Schottky plot near 1.6 V suggests that a portion of the defects can be further oxidized.^39,40^ Consistent with the analysis above, the most suggested form of localized donors within the bandgap of tin oxide is oxygen vacancies.^41–44^ Furthermore, oxygen vacancies can trap one or two free electrons.^27,30^ An oxygen vacancy with one trapped electron has paramagnetic behavior due to the presence of an unpaired electron,^28^ and it can be directly detected using EPR spectroscopy as shown in Figure 1D. Notably, EPR is a technique that directly detects unpaired electrons in a material by measuring the resonant absorption of microwave radiation in the presence of a magnetic field.

Oxygen vacancies in tin oxide lead to the presence of substoichiometric Sn­(II) cations alongside Sn­(IV). The concentration of these Sn­(II) sites and oxygen vacancies can be tuned through the synthesis conditions: high-temperature calcination tends to minimize defects, whereas partial reduction increases them.^27,28,34^ In previous work on doped tin oxide, we demonstrated that higher conductivity correlates with lower charge-transfer resistance and enhanced electrochemical activity.^3^ Here, we show that the same trend applies to undoped tin oxide. To investigate how these nonidealities affect water oxidation activity, we prepared tin oxide under three distinct conditions: fully calcined at 1000 °C (most oxidized, fewest oxygen vacancies/Sn­(II)), calcined at 250 °C (the primary catalyst in this study), and partially reduced at 250 °C following a 250 °C calcination (most reduced, highest oxygen vacancies/Sn­(II)).

Analysis of these samples revealed clear correlations between the preparation conditions and material properties. The Sn 3d XPS spectra in Figure 2A exhibit a binding energy blue shift with increasing oxidation state, suggesting lower Sn­(II)/O_vac_ concentrations in more oxidized samples. We note that distinguishing Sn^2+^ from Sn^4+^ using XPS is inherently challenging, so these assignments are suggestive rather than definitive.^45^ Nevertheless, the XRD patterns in Figure 2B support these observations, showing that all catalysts maintain the tetragonal SnO_2_ crystal structure rather than forming separate crystalline SnO or metallic Sn phases. It should be noted, however, that amorphous SnO or metallic Sn, if present, would not be detectable by XRD. Concurrently, EIS at open-circuit potential in Figure 2C demonstrates that partial reduction significantly lowers charge-transfer resistance, whereas higher-temperature calcination increases it. Four-point probe measurements in Table 1 also show that the electrical resistivity of the catalysts correlates directly with the observed charge-transfer resistance, where partial reduction increases the catalyst conductivity and further oxidation decreases it, which is consistent with trends in the literature.^39,40^ More importantly, more conductive tin oxide catalysts have higher electrochemical performance at a given working potential, as evidenced by the CV results in Figure 2D. These trends are consistent with our prior work on doped tin oxides, which showed a similar relationship between electrical conductivity, charge transfer resistance, and catalytic activity.^3^ Despite the superior performance of tin oxide calcined at 250 °C relative to 1000 °C, neither condition achieves satisfactory water oxidation activity due to relatively high charge transfer resistance, highlighting why undoped tin oxide is rarely used as a standalone catalyst. Taken together, Figure 2A–D suggest that increasing Sn­(II) content directly enhances activity.

**Figure 2:**
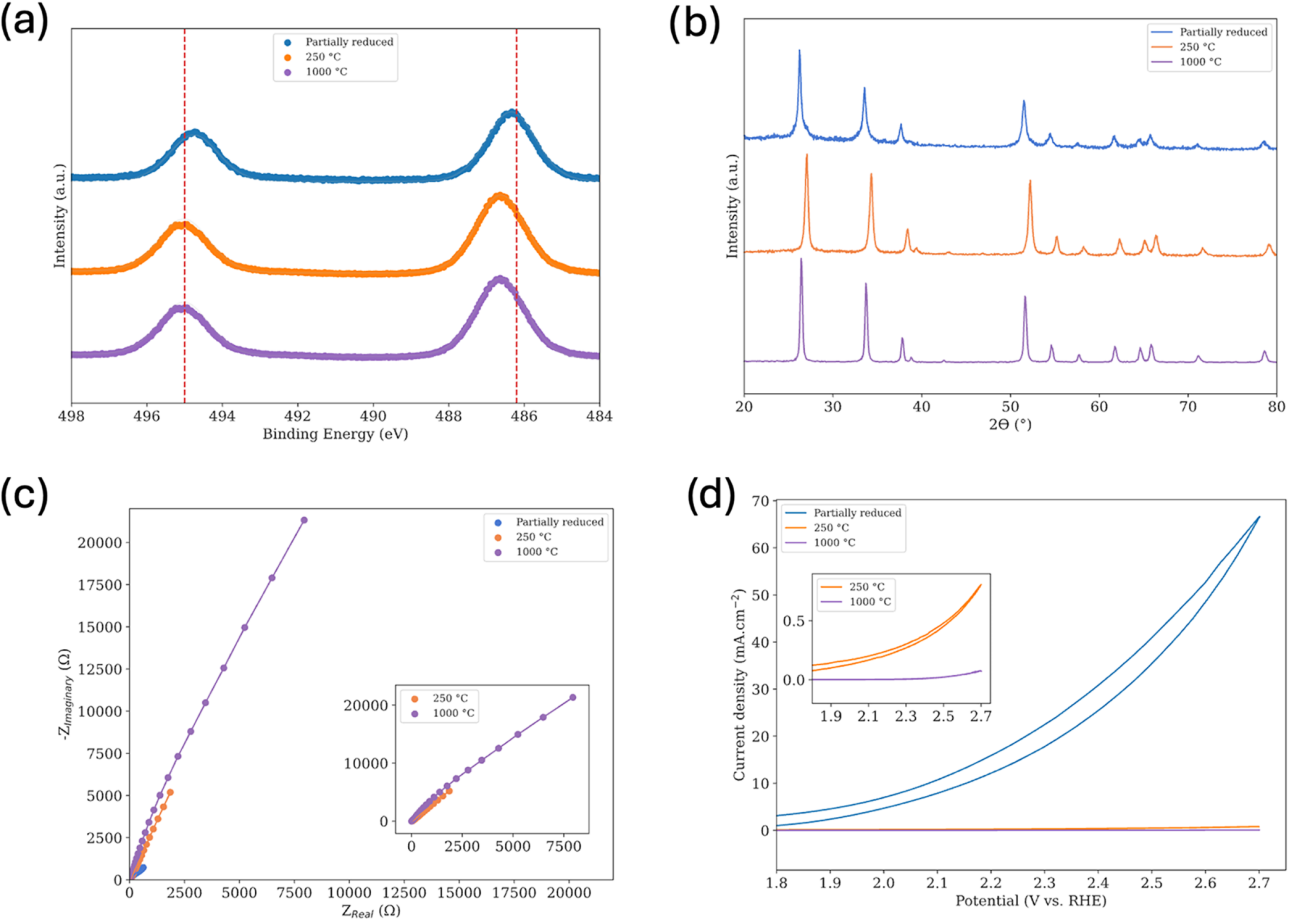
(a) Sn 3d XPS spectra of the different tin oxide catalysts, showing a progressive binding energy blue shift with increasing oxidation state. (b) XRD patterns of all catalysts, confirming the rutile SnO_2_ crystal structure and the absence of crystalline SnO or metallic Sn phases within detection limits. (c) Nyquist plots from EIS measured at OCP in 1 M H_2_SO_4_, illustrating the effect of reduction and calcination on charge transfer resistance (inset highlights the low-impedance region). (d) CVs recorded in 1 M H_2_SO_4_ at 75 mV s^–1^, comparing electrochemical activity across samples (inset expands the low-current region for clarity).

Our density functional theory (DFT) investigations, detailed in the SI, reveal that the SnO_2_(110) surface behaves far from ideally, with intrinsic nonidealities playing a central role. Specifically, water at the interface does not act as a passive medium but undergoes extensive dissociation, generating a hydrogen-bonded network of surface adsorbates (*OH, *H). These adsorbates form what we term “interfacial networks,” which can induce local proton activity that deviates substantially from the bulk electrolyte. This atomistic picture provides a natural framework for linking catalytic activity to corrosion in tin oxide.^5,9^ Two possibilities arise: (i) increased catalytic activity, promoted by defects and *OH/*H networks, may enhance ROS formation, accelerating corrosion, or (ii) the structuring of interfacial proton networks may generate localized acidity (pH drops) at the catalyst–electrolyte interface that destabilizes the oxide lattice.

This latter explanation is strongly supported by recent experimental findings. Li et al. demonstrated with Au micro- and nanoelectrodes in SECM that OER is accompanied by substantial local acidification, with proton concentrations near the IrO_2_ catalyst surface reaching up to 16 M under anodic polarization, even in a nominally dilute perchloric acid electrolyte.^31^ These results confirm that severe deviations between the bulk and interfacial pH are possible during water oxidation. Moreover, these observations align directly with our DFT-derived picture of interfacial proton networks and highlight that local acidity may play a decisive role in driving SnO_2_ corrosion.

On the other hand, Cachet et al.^22^ previously proposed an ROS-driven mechanism, suggesting that electrophilic ROS species attack electron-rich centers within SnO_2_, such as Sn­(II) sites and O_vac_, thereby accelerating corrosion. To test this hypothesis, we introduced hydroxybenzoic acid (HBA), a phenolic compound, into the electrolyte at varying concentrations. HBA was chosen for its known ROS-scavenging properties.^3,10,46^ If ROS plays a critical role in the corrosion process, increasing the concentration of HBA in the electrolyte should scavenge more ROS before they can interact with and degrade the catalyst. This scavenging and corresponding catalyst stabilization should manifest as lower concentrations of dissolved tin species in the electrolyte, which can be detected by using ICP-MS. Figures 3A shows CVs with varying concentrations of HBA in a 1 M H_2_SO_4_ electrolyte. We note however that the addition of HBA introduced a considerable amount of noise to our data, which manifested as large error bars in our results. Nevertheless, the current density clearly decreases with HBA concentration. To deconvolute ROS scavenging from lower electrochemical activity, the total amount of tin detected was normalized by the total Coulombic charge passed during electrolysis. This normalization accounts for the reduced activity and provides a more accurate measure of HBA’s effectiveness in mitigating catalyst degradation through ROS scavenging. Dissolution measurements in Figure 3 B were carried out under galvanostatic conditions at 2.7 V vs RHE in 1 M H_2_SO_4_ and demonstrate that the addition of a scavenger does not stabilize tin oxide during water oxidation. These results, taken together, suggest that ROS attack is not the primary mechanism governing tin oxide corrosion under our conditions. Consequently, although ROS-driven corrosion pathways have strong precedent in the literature, our findings strongly indicate that ROS attack is unlikely to be the dominant mechanism for SnO_2_ dissolution, pointing instead toward alternative pathways such as interfacial proton-network-induced acidity. While this is a logical pathway, our combined DFT, electrochemical, and ICP-MS data suggest that interfacial acidification is more important.

**Figure 3:**
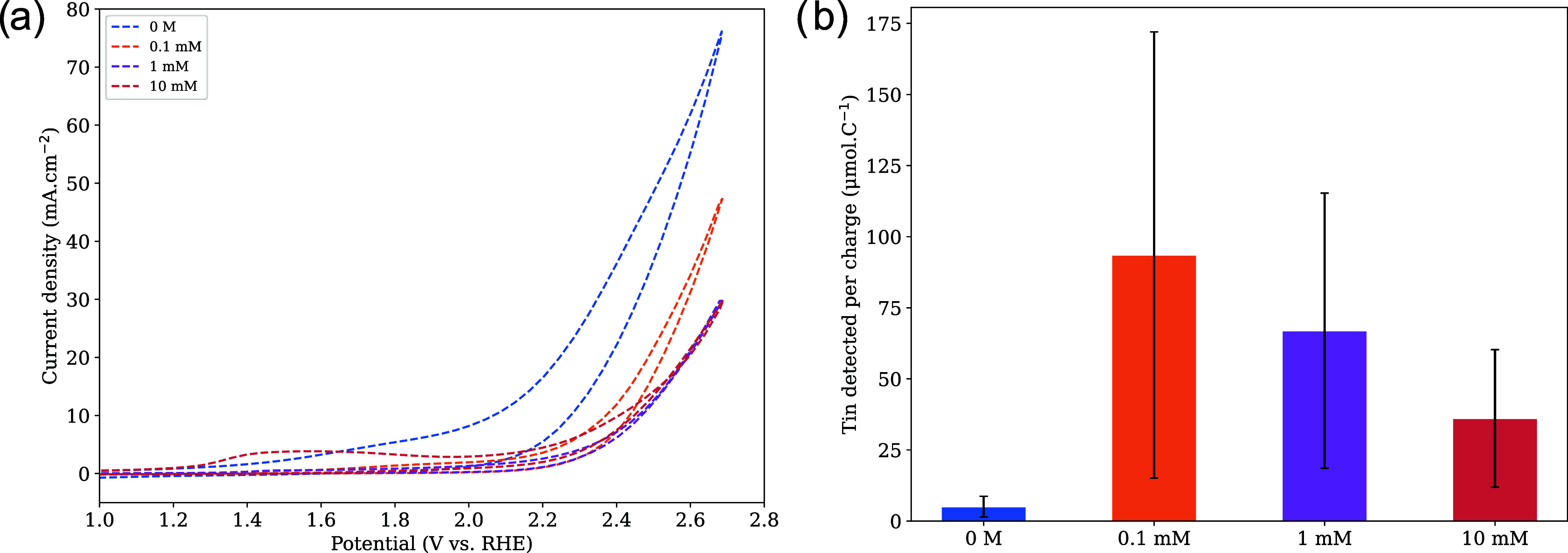
(a) Cyclic voltammograms recorded at a scan rate of 75 mV s^–1^ in 1 M H_2_SO_4_ with varying concentrations of HBA, highlighting the effect of additive concentration on the electrochemical response. (b) Quantification of dissolved tin species determined by ICP-MS at 2.7 V vs RHE, normalized to the total charge passed during electrolysis, providing a measure of tin dissolution per unit of charge.

To further investigate corrosion, we employed a rotating ring-disk electrode (RRDE) setup with a glassy carbon disk and a ring configuration. This was coupled to square-wave voltammetry (SWV) to monitor corrosion under dynamic conditions. In SWV, a square-wave pulse is applied to the top of a staircase potential. At each step, the current at the peak of the pulse is subtracted from the current at the lowest point of the pulse. This differential measurement effectively cancels out nonfaradaic (capacitive) current, enhancing the detection of faradaic processes.^47^ We note that traditional RRDE configurations have been previously used to monitor ROS generation and electrode corrosion.^39,48^ However, when we employed similar configurations using glassy carbon, platinum, and gold rings, we were unable to detect any products, as shown in the Supporting Information Figure S3.

Our approach leverages a two-stage electrochemical process on the collector ring to identify and quantify tin species released from the corroding tin oxide on the disk, where an initial electrodeposition step is employed for increasing the analytical signal to detectable levels. This initial accumulation phase is necessary because corrosion processes release only very small, dilute amounts of tin cations into the bulk solution, which is otherwise too low for direct measurement, as demonstrated by Figure S3. The nature of this signal-enhancing electrodeposition is carefully controlled by the applied potential to differentiate between tin oxidation states. Specifically, Sn­(IV) cations, once generated, are collected on the ring by applying a cathodic potential, causing their reduction to metallic tin. In contrast, dissolved Sn­(II) cations have two collection pathways: they can also be cathodically electroplated as metallic tin or, distinctively, they can be collected by applying an anodic potential to the ring. In this latter anodic process, Sn­(II) is oxidized to Sn­(IV), which then readily hydrolyzes and precipitates onto the ring as tin­(IV) oxide (SnO_2_).

Following this selective electrodeposition, a SWV is applied to the ring. This serves to confirm the presence and quantify the amount of deposited tin, which is now sufficiently concentrated to yield a clear signal. SWV works by sweeping the potential in the direction opposite to the initial deposition. For instance, if Sn­(IV) species were initially collected as metallic tin via cathodic electroplating, a subsequent anodic SWV sweep would oxidize this metallic Sn, producing a characteristic current signal. Similarly, if Sn­(II) was collected as metallic tin through cathodic electroplating, it too will be detected by its anodic oxidation during SWV. However, if Sn­(II) was collected as SnO_2_ via anodic electroprecipitation, a cathodic SWV sweep is used to reduce the SnO_2_, again generating a quantifiable signal. This combination of controlled electrodeposition to first accumulate and differentiate, thereby boosting the signal, followed by SWV for sensitive detection of the reverse redox process, provides a robust method to identify the specific oxidation states of tin species released during corrosion of the tin oxide disk. Scheme 1 summarizes our method. We also note that only the catalyst calcined at 250 °C was used for this part.

**Scheme 1:**
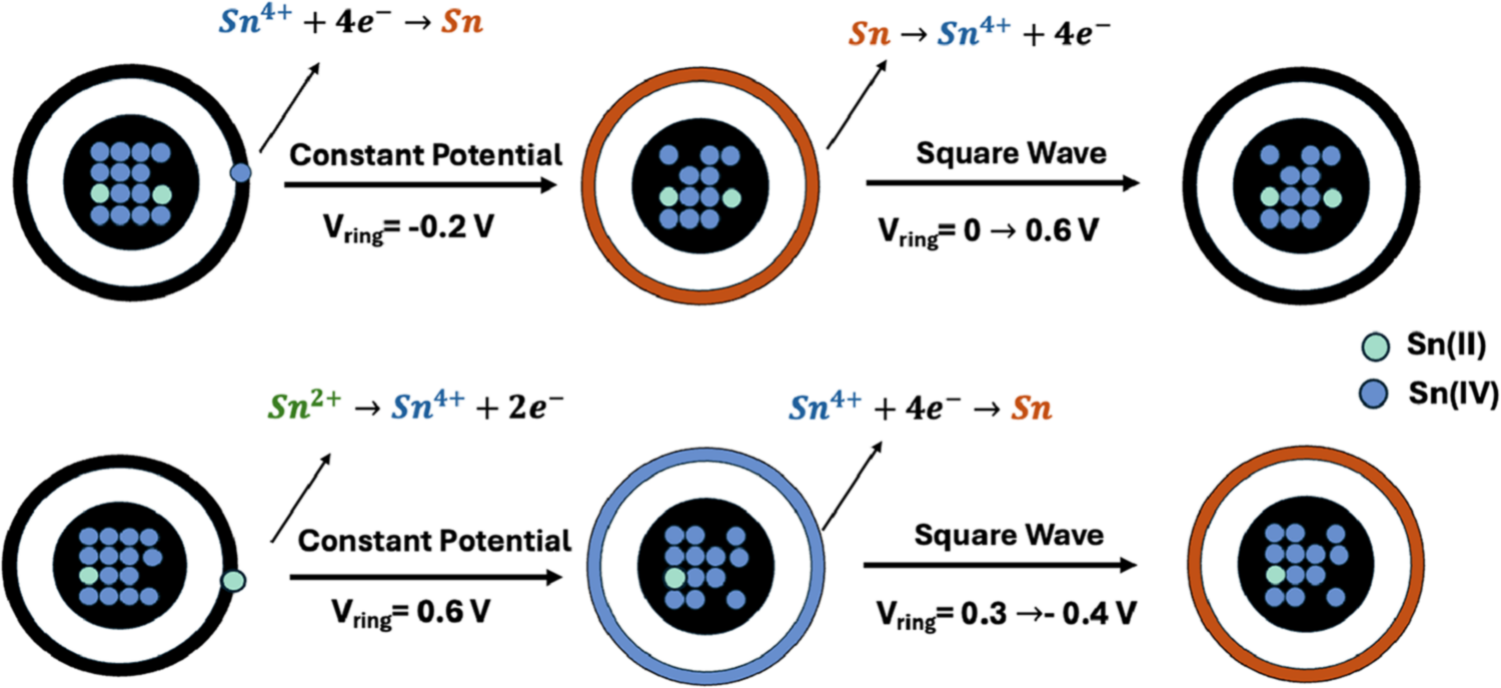
Illustration of the Detection of Different Tin Cations

Figure 4 illustrates the chemical dissolution products collected and detected at the ring when no potential was applied at the disk. As shown in Figure 4A, only Sn­(IV) was detected as the cathodic square wave failed to detect Sn­(II), as evident in Figure 4b. These findings suggest that, as expected, chemical dissolution of tin oxide releases predominantly Sn­(IV) cations into the solution. These results do not negate the feasibility of Sn­(II) dissolution from nonideal tin oxide, but they rather indicate that the rate of Sn­(II) dissolution under the tested conditions is negligible in comparison to that of Sn­(IV), which is consistent with the catalyst composition.

**Figure 4:**
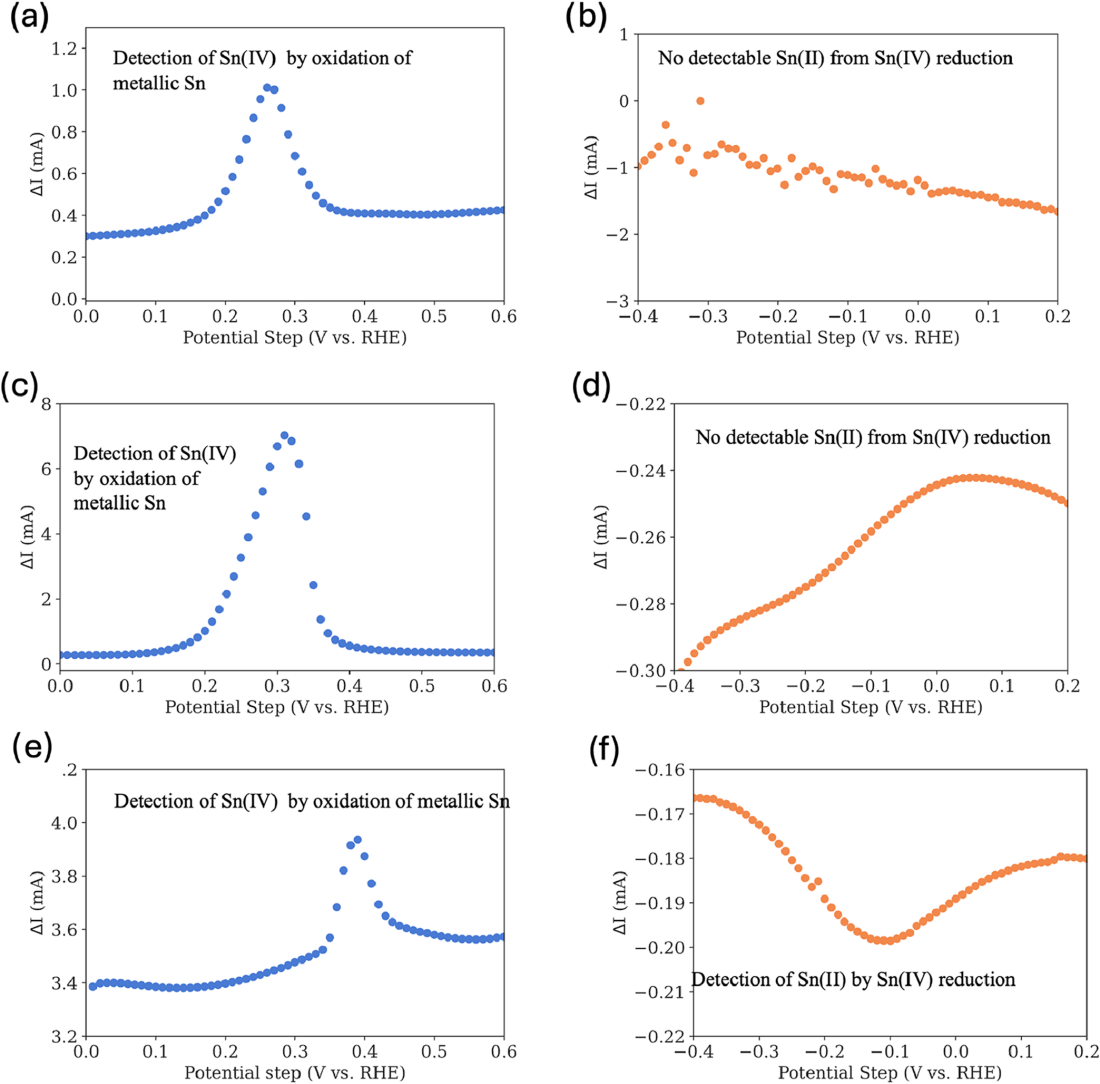
Detection of chemical and galvanic corrosion products under different electrochemical conditions. (a) At open-circuit potential (OCP), Sn­(IV) is detected, while (b) no Sn­(II) signal is observed. Under galvanostatic conditions at a constant potential of 2.7 V vs RHE, (c) Sn­(IV) is detected, whereas (d) Sn­(II) remains undetected; the minor fluctuations in background current are attributed to experimental noise. When the disk is pulsed between 2.7 V and the OCP, both (e) Sn­(IV) and (f) Sn­(II) are detected.

Subsequently, a constant potential of 2.7 V vs RHE was applied to the disk while corroded tin cations were electrodeposited at the ring through either oxidation or reduction. As illustrated in Figure 4C and 4D, the detection of tin cations occurred exclusively via an oxidation square wave, confirming that Sn­(IV) is the sole oxidation state of tin reaching the ring under these experimental conditions. As under an open-circuit potential, no Sn­(II) was detected during constant potential oxidation. It is essential, however, to interpret these findings within experimental limitations. The oxidation potential applied at the ring is significantly lower than that at the disk (0.6 V at the ring compared to 2.7 V at the disk), and the geometric area of the ring is smaller than that at the disk. Consequently, any Sn­(II) species generated at the disk is highly likely to oxidize to Sn­(IV) directly at the disk surface before diffusing to the ring. This dynamic renders the detection of Sn­(II) at the ring highly unlikely under constant potential conditions. The results also suggest that under constant polarization, any Sn­(II) will be oxidized to Sn­(IV) before it can be detected. Therefore, to detect Sn­(II) cations, the disk potential was pulsed between the OCP and 2.7 V vs RHE, while the ring potential remained constant at 0.6 V vs RHE. We rationalized that Sn­(II) generated during the pulse would diffuse to the ring instead of being oxidized at the disk during the OCP period. As shown in Figure 4E, Sn­(IV) was detected anodically, which confirms that corrosion still occurs under pulse conditions. More importantly, Figure 4F reveals that Sn­(II) is also detected at the ring by using a cathodic square wave, confirming its presence among the corrosion products. We note that when the catalyst calcined at 1000 °C was used with the pulsed method, no Sn­(II) was detected. In summary, our analysis demonstrates that both Sn­(IV) and Sn­(II) corrode from tin oxide during water oxidation.

Our findings on the role of interfacial water and its potential effects on interfacial pH reduction and the corrosion of Sn­(II) and Sn­(IV) suggest a complex interplay between corrosion and catalysis. This interplay may explain many experimental observations regarding tin oxide and other metal oxides. First, interfacial pH reduction, which is caused by interfacial water adsorption as shown in the SI, is likely responsible for the accelerated corrosion of both Sn­(II) and Sn­(IV) cations, leading to faster catalyst degradation

1 $$2H_{2}O\to O_{2}+4H^{+}+{4e}^{-}$$

2 $$Sn_{x}^{\left(\mathrm{IV}\right)}Sn_{y}^{\left(\mathrm{II}\right)}O_{2x+y,\left(s\right)}+{2H}_{\left(\mathrm{aq}\right)}^{+}\to {\mathrm{Sn}}_{\left(\mathrm{aq}\right)}^{2+}+Sn_{x}^{\left(\mathrm{IV}\right)}Sn_{y-1}^{\left(\mathrm{II}\right)}O_{2x+\left(y-1\right),\left(s\right)}{+H}_{2}O$$

3 $$Sn_{x}^{\left(\mathrm{IV}\right)}Sn_{y}^{\left(\mathrm{II}\right)}O_{2x+y,\left(s\right)}+4H^{+}\to {Sn}_{(\mathrm{aq})}^{4+}+Sn_{x-1}^{\left(\mathrm{IV}\right)}Sn_{y}^{\left(\mathrm{II}\right)}O_{2\left(x-1\right)+y,\left(s\right)}{+2H}_{2}O$$

Here Sn_*x*_^(IV)^Sn_*y*_^(*II*)^O_2*x*+*y*,(*s*)_ represents a nonideal tin oxide catalyst, with *x* Sn­(IV) units and *y* Sn­(II) units on its surface. Therefore, a more active catalyst would induce a greater pH reduction, which would lead to faster catalyst degradation. This phenomenon has been corroborated in the literature.^40,49^

Second, the absence of detectable Sn­(II) at the ring during constant potential electrolysis at the disk suggests that once Sn­(II) cations corrode, they can be redeposited as Sn­(IV) within the catalyst structure. This process likely follows a three-step mechanism: An interfacial decrease in pH due to increased proton release during water oxidation (eq 1); Chemical dissolution of Sn­(II), driven by the interfacial pH drop (eq 2); Electrochemical redeposition of Sn­(II) as Sn­(IV), accompanied by the incorporation of two water-derived oxygen atoms into the tin oxide lattice to maintain charge neutrality (eq 4).

4 $${\mathrm{Sn}}_{\left(\mathrm{aq}\right)}^{2+}+2{\mathrm{OH}}_{(\mathrm{ads})}+Sn_{x}^{\left(\mathrm{IV}\right)}Sn_{y-1}^{\left(\mathrm{II}\right)}O_{2x+\left(y-1\right),\left(s\right)}\to Sn_{x+1}^{\left(\mathrm{IV}\right)}Sn_{y-1}^{\left(\mathrm{II}\right)}O_{2\left(x+1\right)+\left(y-1\right),\left(s\right)}+{2H}_{(\mathrm{aq})}^{+}$$

Finally, our findings also provide insight into the formation of products originating from the oxygen lattice, a process previously linked to irreversible corrosion.^15,17^ This phenomenon can be explained by a two-step mechanism: (1) Chemical dissolution of either Sn­(II) or Sn­(IV) (eqs 2 and 3), leading to the release of lattice oxygen as water, and (2) Electrochemical oxidation of the released water, generating products derived from lattice oxygen (eq 1).

## 4 Conclusion

This study elucidates the complex interplay among the structural, chemical, and electrochemical properties of tin oxide catalysts, offering novel insights into their activity, stability, and lattice oxygen behavior. The presence of substoichiometric Sn­(II) within the tin oxide structure greatly enhances electrical conductivity and electrocatalytic activity, yet it also increases corrosion due to proton adsorption and interfacial pH reduction during enhanced electrochemical water oxidation. These findings underscore the inherent trade-off between activity and stability, as more active catalysts with a higher Sn­(II) content are less stable due to accelerated corrosion. The study also reveals distinct dissolution behaviors for Sn­(II) and Sn­(IV). While only Sn­(IV) is detected during chemical dissolution, both Sn­(II) and Sn­(IV) are observed during galvanic corrosion. A proposed dynamic mechanism involving the dissolution of Sn­(II), its redeposition as Sn­(IV), and the reintegration of water oxygen into the lattice highlights the irreversible nature of these processes and clarifies the origin of lattice oxygen in water oxidation products. A two-step process comprising the chemical dissolution of Sn­(II) or Sn­(IV) and the subsequent electrochemical oxidation of water released during dissolution explains the release of lattice oxygen as products independent of direct lattice oxygen activity. The proposed reaction schemes suggest that lattice oxygen’s involvement in water oxidation may be a side effect of localized corrosion rather than an entirely separate catalytic role.

## Supplementary Material



## Notes

Density functional theory (DFT) methods and resulting free energy and phase diagrams; rotating ring-disk electrode voltammetry; square-wave voltammetry reference curves (PDF)
