# Supplementary material for: Electrochemical Corrosion and Catalysis Dynamics of Tin Oxide during Water Oxidation
Source: ACS Catal. 2025 Oct 28;15(21):18601–11. doi: 10.1021/acscatal.5c04461 (PMC12603990; doi:10.1021/acscatal.5c04461)
Supplement: Supplementary file 1 [file cs5c04461_si_001.pdf]

Supporting Information for Electrochemical Corrosion and Catalysis Dynamics of Tin Oxide During Water Oxidation

Rayan Alaufey[a,], Lingyan Zhao[b], Christina Lents[a], Brianna Markunas[a], Adam D. Walter [c], Qin Wu[f]d, John A. Keith[b], and Maureen Tang*[a]

[a] Department of Chemical and Biological Engineering, Drexel University, Philadelphia 19104, Pennsylvania, United States

[b] Department of Chemical and Petroleum Engineering, University of Pittsburgh, 3700 O'Hara Street, Pittsburgh, PA 15261

[c] Department of Material Science and Engineering, Drexel University, Philadelphia 19104, Pennsylvania, United States

[d] Center for Functional Nanomaterials, Brookhaven National Laboratory, Upton, NY, 11973, USA

mhtang@drexel.edu

Table of Contents

1. Computational Methods **2**

2. Theoretical Insights into Non-Idealities and Interfacial Water Effects **3**

3. Traditional Rotating Ring-Disk Electrode Analysis **8**

4. Square wave voltammetry controls with tin chloride **9**

5. References **10**

1. Computational Methods

In previous work, we employed a 2 × 1 SnO_2(110) surface slab model to study reaction steps. In this study, we expanded the model to a 2 × 2 SnO_2(110) surface slab to more accurately represent adsorbate configurations relevant to potential reaction pathways.[1,2] All calculations were performed using Kohn–Sham density functional theory (DFT) as implemented in the Vienna Ab initio Simulation Package (VASP) version 5.4.4 with GPU acceleration.[3–6]

Electronic energies were computed using the Perdew-Burke-Ernzerhof (PBE) exchange-correlation functional with Projector Augmented Wave (PAW) pseudopotentials and a plane-wave energy cutoff of 450 eV. Spin polarization was included to model the triplet state of the O_2 molecule. A 4×8×1 Monkhorst-Pack k-point grid was used for all surface calculations. The bottom two layers of the slab were fixed, while the upper two layers and adsorbates were allowed to relax until the energy change between steps was less than 0.1 meV, using the conjugate gradient algorithm.

For all slab models, the absolute free energy of each system was approximated as the DFT electronic energy calculated using VASP, assuming negligible contributions from zero-point vibrational and thermal effects. For gas-phase water and product molecules used in conjunction with slab calculations, we combined DFT electronic energies with thermodynamic entropy contributions obtained from the NIST Webbook**.**[7] For solvated water, we added solvation energy contributions, including appropriate standard state corrections, to the gas-phase DFT energy, following established protocols.[8] These data were integrated with previously reported solution-phase reactive oxygen species (ROS) energies calculated using high-level methods.

Computational Hydrogen Electrode (CHE) Model and Reaction Pathway Analysis

The Computational Hydrogen Electrode (CHE) model and electrochemical surface phase diagrams were used to analyze reaction intermediates at different applied potentials.[9,10] To study water oxidation tin oxide, we consider 6 potential reaction steps:

1. $H_2O + * \rightarrow OH^* + H^+ + e^-$
2. $OH^* \rightarrow O^* + H^+ + e^-$

3. $O^* + H_2O \rightarrow OOH^* + H^+ + e^-$

4. $OOH^* \rightarrow O_2^* + H^+ + e^-$

5. $O_2^* + H_2O \rightarrow HO_3^* + H^+ + e^-$

6. $HO_3^* \rightarrow O_3 + * + H^+ + e^-$

Here, * denotes a surface adsorption site, and O* represents an oxygen atom adsorbed on the surface. These equations are applicable to both the adsorbate evolution mechanism (AEM) and the lattice oxygen mechanism (LOM)**,** with the distinction lying in whether the oxygen atoms originate from the water (AEM) or from the generation of oxygen vacancies in the oxide lattice (LOM).

2. Theoretical Insights into Non-Idealities and Interfacial Water Effects

Experimental results strongly support the presence of oxygen vacancies that also have a direct effect on tin oxide activity. Previous theoretical studies have suggested that oxygen vacancies play a direct role in water oxidation, facilitating the production of both oxygen and ozone via the lattice oxygen mechanism (LOM), which involves the direct coupling of lattice oxygen atoms to form products. Our prior work investigated this possibility and demonstrated that the LOM pathway is less favorable than the more conventional adsorbate evolution mechanism (AEM).[2,11] We now present results for LOM on $SnO_2(110)$ using DFT calculations with a 2×2 unit cell, both with and without explicit water co-adsorbates. In Figure S1, we examine the energetics of oxygen migration to the surface, creating a vacancy on a clean $SnO_2(110)$ surface in the presence of various adsorbates, including one or more explicit water molecules.

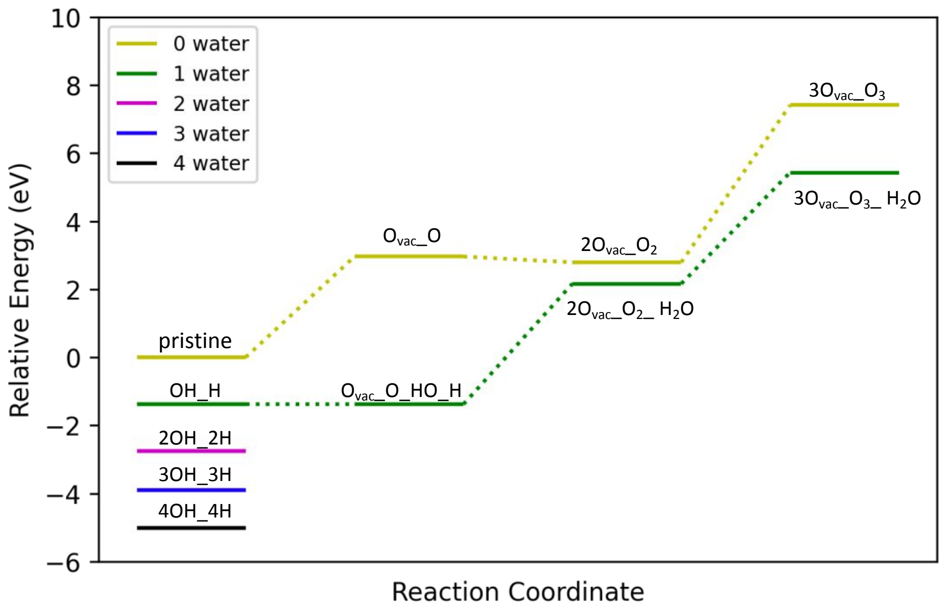

Figure S1: A calculated free energy diagram illustrates the relative energetics of LOM processes and the impact of explicit water on the surface. The x-axis represents the sequence of chemical steps leading to ozone production. The yellow line corresponds to LOM steps without coadsorbed water, while the green line represents LOM steps with one explicit water molecule occupying the oxygen vacancy site. The purple, blue, and black lines indicate the relative energies of two, three, and four dissociated water molecules adsorbed on the surface. Calculations for additional vacancies were not performed. The net effect is that water molecules are seen to significantly stabilize the adsorbed intermediates and fill surface oxygen vacancies.

The steps corresponding to the yellow line begin with a bridging oxygen atom on SnO_2(110) migrating to an Sn top site, creating a surface oxygen vacancy (Ovac_O). This step is calculated to be energetically uphill by +2.96 eV. The top-site oxygen adsorbate then binds with another bridging oxygen, forming an O_2 adsorbate (2Ovac_O_2), which is slightly lower in energy by 0.20 eV relative to the Ovac_O state. The O_2 intermediate can further interact with an in-plane oxygen, leading to a state that is 4.65 eV higher than the 2Ovac_O_2 state. Our calculated oxygen vacancy formation energy on the 2 × 2 SnO_2(110) surface (+2.17 eV) aligns reasonably well with our previous calculations on the 2 × 1 unit cell (+2.30 eV) as well as other reliable literature values, which fall within the range of 2.0–2.3 eV.[2,12] We then modeled an analogous mechanism involving a coadsorbed water molecule, represented by the green line in Figure S1. Initially, a single water molecule binds to SnO_2(110) with an energy of 1.38 eV after dissociating into O* and OH* intermediates. This observation aligns with previous calculations by Santarossa et al.,

who used Born-Oppenheimer molecular dynamics to study water adsorption on the $SnO_2(110)$ surface.[13] By comparing the energetics of the first step in the yellow line pathway with the first step in the green line pathway, we find a significant driving force (4.34 eV) for refilling the oxygen vacancy with an explicit water molecule. Additionally, a single water molecule provides stabilization energies of 0.63 eV for O_2* adjacent to two oxygen vacancies (one occupied by water) and 1.97 eV for O_3* next to three oxygen vacancies (one occupied by water). As a result, the reaction energy for O_2 forming O_3 via a lattice oxygen atom decreases from +4.61 eV to +3.27 eV due to the presence of explicit water.

When a second water molecule is added to the surface, it dissociates, resulting in a surface covered with dissociated water molecules represented by the pink line. The adsorption energy of two dissociated water molecules on $SnO_2(110)$ is −2.78 eV, which is approximately 0.85 eV more stable than configurations with one dissociated and one intact water molecule or two intact water molecules. When three and four water molecules bind to the surface, all are found to dissociate, with binding energies of −3.90 eV and −5.02 eV, respectively. These results indicate that on $SnO_2(110)$, water adsorption energy follows a nearly linear trend until the surface coverage reaches approximately 75%, suggesting minimal steric hindrance among dissociated water molecules below this threshold. This also indicates that the lowest energy facet of tin oxide under ambient electrochemical conditions is highly unlikely to resemble a clean surface, but they will rather be a surface covered with OH* and H* intermediates, which in turn would also likely interact with non-adsorbed water molecules. Based on these results, calculations involving a limited number of explicit hydrogen bonding interactions on the surface should provide insights into water oxidation mechanisms.

As an initial assessment of which surface adsorbate structures are the most energetically viable at different electrode potentials, we compiled 48 different geometrically relaxed structures on the undoped and clean slab systems that involved up to four oxygen atoms and four hydrogen atoms, all into an electrochemical atomistic thermodynamics model. In this way, we could model the following putative intermediates: OH*, O*, OOH*, O_2*, O_3H*, and O_3*, all in the presence of different numbers of hydrogens and oxygens that would arise from co-adsorbed species. We then categorized all the surface structures based on the number of oxygen and hydrogen atoms they contained, and then we identified the lowest energy structure for each category (detailed in the

SI) and illustrated it using figures to the right of the phase diagram. The *x*-axis shows a change in chemical potential of bulk water. The vertical dashed line is set to $\Delta\mu_{H_2O} = 0$, i.e., the state obtained directly from the DFT calculations with no other thermochemical approximations.

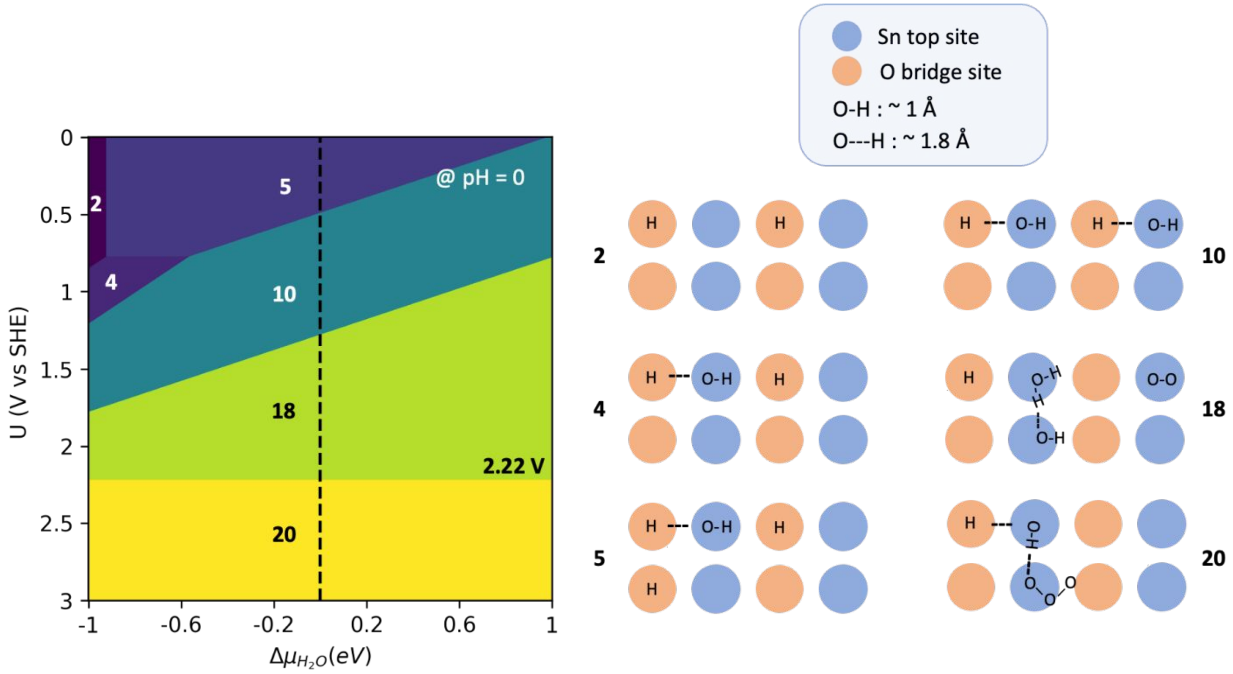

Figure S2: An electrochemical phase diagram representing the stability of different atom configurations on an undoped pristine $SnO_2(110)$ surface as a function of chemical potential of water, μ_{H_2O}, and applied potential at pH = 0. On the right, surface structures are depicted using orange dots that represent bridging oxygen sites and blue dots represent tin active sites on $SnO_2(110)$.

Figure S2 shows the most stable structures at pH 0 at different applied potentials and chemical potentials of water. The region denoted with the label of 20 shows that the most stable surface structure in the range of applied potentials higher than 2.22 V is O_3* co-adsorbed with dissociated water. The diagram also shows that the thermodynamic limiting potential of the pristine surface is 2.22 V. The diagram further illustrates that under almost all conditions, except those denoted using label 2, where chemical potentials of water are more negative than −0.9 eV

and applied potentials are less than 1 V, at least one water molecule will co-adsorb onto the surface in a dissociative manner. In fact, based on Figure S2, considerations of additional water molecules would presumably allow up to full surface coverage of adsorbed water. However, it is unclear whether additional co-adsorbed waters at other surface sites would change the qualitative aspects of this analysis since: (1) we observed a roughly linear correspondence in the sequential binding energies of water on this surface, indicating low levels of interactions between adsorbates bound to different Sn sites, and (2) modeling each surface with a fully saturated layer of water is limited by the fact that species 18 has one vacant Sn site while species 20 has two vacant sites. Note that our model assumes that all oxygen atoms are indistinguishable and that additional oxygen atoms can be readily obtained from solution-phase water. In this way, we anticipate a degree of error cancellation when comparing relative energies. Our present model indicates that the most thermodynamically stable surface structure at high applied potentials prior to forming O_2* consists of two co-adsorbed water molecules that are dissociated across multiple sites. Lastly, our figure shows that the water oxidation potential would not be significantly impacted by the chemical potential of water because structure 18, which represents O_2* co-adsorbed with two water molecules, has the same number of oxygen atoms as structure 20 which represents O_3* co-adsorbed with one explicit water.

3. Traditional Rotating Ring-Disk Electrode Analysis

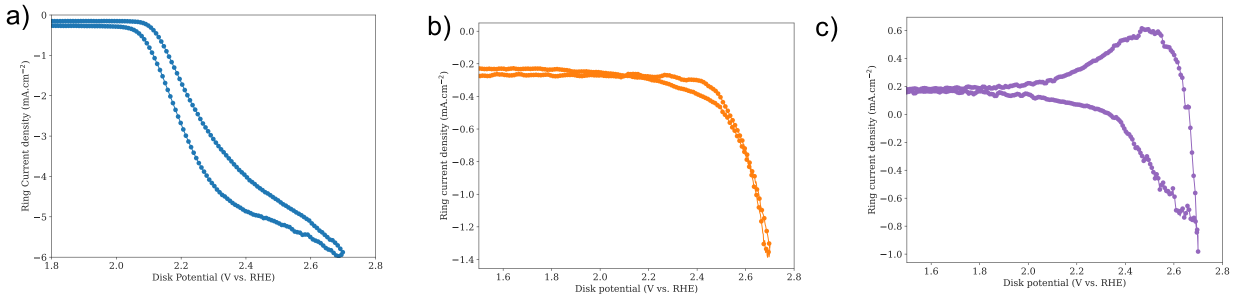

Figure S3: Rotating Ring-Disk Electrode (RRDE) Assessment of Tin Species During Water Oxidation on Tin Oxide. The tin oxide-coated glassy carbon disk was cycled to 2.70 V vs. RHE, with the ring (a) Pt, b) Au, or c) glassy carbon) held at -0.3 V vs. RHE (1600 rpm, Ar purge).

While the onset of faradaic oxygen reduction at the ring electrode clearly varied depending on the ring material, occurring at a disk potential of 2.15 V for Platinum, 2.4 V for Gold, and 2.5 V for Glassy Carbon no corresponding signal for corroded tin species (Sn(IV) reduction) was detected on any of the ring materials**.** This absence of detectable tin is attributed to the inherently limited sensitivity of this conventional RRDE method for trace analyte detection, especially when compared to the potentially low flux of dissolved tin species. Consequently, these results underscore the critical need for more sensitive electrochemical techniques, such as square-wave voltammetry (SWV) coupled with a pre-accumulation step at the ring electrode, to reliably detect and quantify any tin corrosion products generated during the anodic processes.

4. Square wave voltammetry controls with tin chloride

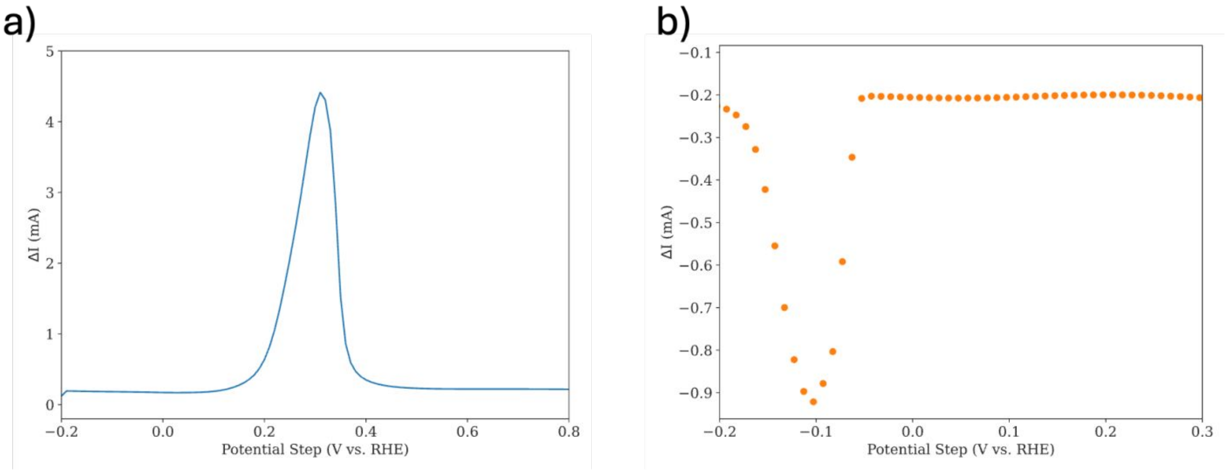

Figure S4: Establishing Reference Square Wave Voltammograms for Tin(IV) and Tin(II) Species: Control SWV measurements were conducted using a glassy carbon ring electrode in Ar-purged 1 M hydrochloric acid. The electrolyte contained either a) $SnCl_4$ to characterize Sn(IV) response, or b) $SnCl_2$ for Sn(II) response.

5. References

(1) Alaufey, R.; Keith, J. A.; Tang, M. A Co-Doping Materials Design Strategy for Selective Ozone Electrocatalysts. *J. Phys. Chem. Lett.* **2024**, *15* (28), 7351–7356. https://doi.org/10.1021/acs.jpclett.4c01150.

(2) Lansing, J. L.; Zhao, L.; Siboonruang, T.; Attanayake, N. H.; Leo, A. B.; Fatouros, P.; Park, S. M.; Graham, K. R.; Keith, J. A.; Tang, M. Gd-Ni-Sb-SnO2 Electrocatalysts for Active and Selective Ozone Production. *AIChE Journal* **2021**, *67* (12), e17486. https://doi.org/10.1002/aic.17486.

(3) Kresse, G.; Hafner, J. *Ab Initio* Molecular Dynamics for Liquid Metals. *Phys. Rev. B* **1993**, *47* (1), 558–561. https://doi.org/10.1103/PhysRevB.47.558.

(4) Kresse, G.; Furthmüller, J. Efficient Iterative Schemes for *Ab Initio* Total-Energy Calculations Using a Plane-Wave Basis Set. *Phys. Rev. B* **1996**, *54* (16), 11169–11186. https://doi.org/10.1103/PhysRevB.54.11169.

(5) Hutchinson, M.; Widom, M. VASP on a GPU: Application to Exact-Exchange Calculations of the Stability of Elemental Boron. *Computer Physics Communications* **2012**, *183* (7), 1422–1426. https://doi.org/10.1016/j.cpc.2012.02.017.

(6) Hacene, M.; Anciaux-Sedrakian, A.; Rozanska, X.; Klahr, D.; Guignon, T.; Fleurat-Lessard, P. Accelerating VASP Electronic Structure Calculations Using Graphic Processing Units. *J. Comput. Chem.* **2012**, *33* (32), 2581–2589. https://doi.org/10.1002/jcc.23096.

(7) Linstrom, P. J.; Mallard, W. G. The NIST Chemistry WebBook: A Chemical Data Resource on the Internet. *J. Chem. Eng. Data* **2001**, *46* (5), 1059–1063. https://doi.org/10.1021/je000236i.

(8) Keith, J. A.; Carter, E. A. Quantum Chemical Benchmarking, Validation, and Prediction of Acidity Constants for Substituted Pyridinium Ions and Pyridinyl Radicals. *J. Chem. Theory Comput.* **2012**, *8* (9), 3187–3206. https://doi.org/10.1021/ct300295g.

(9) *Origin of the Overpotential for Oxygen Reduction at a Fuel-Cell Cathode | The Journal of Physical Chemistry B*. https://pubs.acs.org/doi/10.1021/jp047349j (accessed 2025-03-08).

(10) *Computationally Guided Searches for Efficient Catalysts through Chemical/Materials Space: Progress and Outlook | The Journal of Physical Chemistry C*. https://pubs.acs.org/doi/10.1021/acs.jpcc.0c11345 (accessed 2025-03-08).

(11) Alaufey, R.; Zhao, L.; Lindsay, A.; Siboonruang, T.; Wu, Q.; Keith, J. A.; Wood, E.; Tang, M. Interplay between Catalyst Corrosion and Homogeneous Reactive Oxygen Species in Electrochemical Ozone Production. *ACS Catal.* **2024**, 6868–6880. https://doi.org/10.1021/acscatal.4c01317.

(12) Saravanan, K.; Basdogan, Y.; Dean, J.; Keith, J. A. Computational Investigation of CO2 Electroreduction on Tin Oxide and Predictions of Ti, V, Nb and Zr Dopants for Improved Catalysis. *J. Mater. Chem. A* **2017**, *5* (23), 11756–11763. https://doi.org/10.1039/C7TA00405B.

(13) Santarossa, G.; Hahn, K.; Baiker, A. Free Energy and Electronic Properties of Water Adsorption on the SnO2(110) Surface. *Langmuir* **2013**, *29* (18), 5487–5499. https://doi.org/10.1021/la400313a.
